# Point of Care Testing, Rapid Next Generation Sequencing and Artificial Intelligence in Pediatric and Neonatal Healthcare: A Narrative Review

**DOI:** 10.3390/ph18111721

**Published:** 2025-11-13

**Authors:** Alessandra Cianflone, Luigi Coppola, Pasquale Primo, Giovanna Maisto, Fiorenza Mastrodonato, Maria Antonia Di Palma, Rosanna Parasole, Daniela Omodei, Peppino Mirabelli

**Affiliations:** 1 UOS Laboratori di Ricerca e Biobanca, UOC Ricerca Clinica e Traslazionale, AORN Santobono-Pausilipon, Via Teresa Ravaschieri, n.8, 80129 Naples, Italy; a.cianflone@santobonopausilipon.it (A.C.); l.coppola@santobonopausilipon.it (L.C.); p.primo@santobonopausilipon.it (P.P.); f.mastrodonato@santobonopausilipon.it (F.M.); r.parasole@santobonopausilipon.it (R.P.); p.mirabelli@santobonopausilipon.it (P.M.); 2 Dipartimento di Ematologia, Oncologia e Terapie Cellulari, AORN Santobono-Pausilipon, 80122 Naples, Italy; g.maisto@santobonopausilipon.it; 3 ASL Napoli 3 Sud, 80059 Naples, Italy; 4 Istituto di Biostrutture e Bioimmagini-Consiglio Nazionale delle Ricerche (IBB-CNR), Via De Amicis 95, 80145 Naples, Italy

**Keywords:** pediatrics, children, diagnostics, POCT, NICU, PICU, rapid NGS, artificial intelligence

## Abstract

Laboratory tests play a crucial role in the diagnostic process for both adults and children. Indeed, they are essential for evaluating health status and formulating effective treatment strategies in the presence of disease. However, in the case of pediatrics, distinct physiological and developmental features of children should be taken into account when compared to adults. Consequently, it is necessary to consider some factors, such as reference intervals that vary significantly at different stages of development due to sexual development, cerebral maturation, and biological and environmental influences; furthermore, it must be considered that extremely small volumes of biological samples are often necessary to obtain accurate laboratory results. Finally, timely test results are critical, especially for pediatric conditions that are treatable when diagnosed early. This review article provides a comprehensive overview of advanced diagnostic technologies, including rapid next-generation sequencing and complex point-of-care testing, within the context of pediatric laboratory medicine. Special attention is given to diagnostic tools that support neonatal and pediatric intensive care units, with a focus on how innovative technologies, particularly those utilizing artificial intelligence algorithms, can accelerate diagnostic workflows.

## 1. Introduction

Children are the adults of tomorrow, and safeguarding their health is critically important not only since they are a vulnerable group, but also because early disease detection and intervention can significantly reduce the burden on public health and improve overall societal well-being. To achieve this goal, newborn screening programs have been implemented worldwide, targeting over fifty distinct conditions [1,2]. These initiatives enable the early identification of potential health issues to start life-saving therapies on time. Children and newborns have peculiar physiological and developmental traits that require specialized approaches of diagnosis, treatment, and laboratory testing. This is especially true for neonates, whose unique pathophysiological profile requires special attention [3]. The use of invasive methods to collect vital clinical data is a major problem in neonatal and pediatric diagnostics; in this context, there is an increasing focus on the creation of non-invasive or minimally invasive laboratory tests. Another key challenge lies in the need for rapid turnaround times to ensure timely diagnosis and treatment in neonates and infants. To this aim, developments in laboratory medicine have enabled practical methods called Point of Care Testing (POCT) to measure vital indicators such as blood gases and metabolic markers more quickly and with less discomfort for the child. Also, dried blood spot (DBS) testing, usually collected via heel prick, has significantly improved rapid diagnosis in newborns. As a result, high-throughput technologies have been introduced, allowing laboratories to process large numbers of DBS samples quickly and less invasively. DBS testing is often used in newborn screening programs to identify metabolic or inherited conditions such as biotinidase deficiency (BD), congenital hypothyroidism (CH), galactosemia (GAL), and phenylketonuria (PKU) using mass spectrometry. By simultaneously detecting 40 to 50 metabolic abnormalities from a single blood sample, this technology has transformed the industry and enabled a more thorough assessment of a newborn’s health status [4]. Although there is growing consensus that screening programs should be expanded, the use of such technologies is still limited to a relatively small number of diseases [2]. Furthermore, the scope and coverage of newborn screening programs vary significantly across countries [5,6], influenced by differences in disease prevalence, financial resources, and national healthcare policies. The increasingly low cost of Next-Generation Sequencing (NGS) technology and the expansion of rapid NGS (rNGS) offer promising opportunities to overcome these limitations; indeed, broader implementation of rNGS could enhance diagnostic yield, facilitate earlier interventions, and extend access to diagnostic testing for a wider range of diseases. The sensitivity and specificity of these tests limit DBS screening to a predetermined range of conditions based on biochemical indicators; the identification of a broader spectrum of genetic abnormalities, including rare and ultra-rare conditions not covered by standard panels, is made possible by rNGS, which allows for comprehensive genomic analysis. Furthermore, in emergency settings such as neonatal intensive care units, where early intervention can significantly improve outcomes, the ability of rNGS to provide molecular diagnoses in an exceptionally rapid timeframe is crucial. Furthermore, beyond the binary results of conventional screening, the rapid genetic data also expedite family counseling and personalized treatment plans.

The novel concept of molecular diagnostic POCT by NGS has allowed the introduction for diseases such as sickle cell disease (SCD) and other hemoglobinopathies [7,8], group B Streptococcus [9] and cytomegalovirus [10]. Furthermore, the implementation of NGS in newborn screening programs improves diagnostic performance for cystic fibrosis (CF) [11], severe combined immunodeficiency (SCID) [12] and spinal muscular atrophy (SMA) [13]. In this context, rNGS allows for swift analysis of an individual’s DNA, thus enabling clinicians to make more accurate diagnoses and to implement tailored treatment strategies promptly. Moreover, data obtained from rapid laboratory tests, when analyzed using artificial intelligence (AI)-based diagnostic algorithms, can significantly enhance their clinical utility. The introduction of AI in medicine is revolutionizing healthcare, especially with the creation of diagnostic and prognostic models based on machine learning (ML). These models improve diagnostic accuracy, cost-effectiveness, and speed in clinical laboratories by mining, analyzing, and integrating huge clinical datasets [14], even in the absence of highly trained personnel, as commonly required for traditional bioinformatic analyses [15]. This method improves diagnostic accuracy by enabling deeper analysis of laboratory test results, including new layers of data that would otherwise go undiscovered. Therefore, both rNGS and POCT help broaden the therapeutic window for interventions on genetic and non-genetic diseases, facilitating the rapid identification of life-threatening conditions [16,17]. An additional tool for the treatment of pediatric patients is provided by the integration of AI-based methods into the analysis of molecular and non-molecular POCT data. Indeed, the combination of clinical and epidemiological data provides faster and more accurate diagnoses, as well as better prediction of outcomes. The combined capabilities and synergistic application of rNGS, POCT, and AI would further accelerate clinical interventions and decision-making, which could enhance current screening programs and significantly improve outcomes for critically ill newborns. However, implementing these cutting-edge methods into current clinical practice still presents several challenges. In light of these considerations, the primary objectives of this review are to evaluate the use of rNGS, POCT, and AI, with a particular emphasis on the neonatal and pediatric populations. We will investigate the applications, advantages, limitations, and ethical issues of these technologies, highlighting differences in clinical implementation, challenges, and outcomes between neonatal and general pediatric care.

## 2. Rapid Genome Sequencing for Children’s Diseases

Congenital anomalies due to chromosomal or molecular genetic aberrations are the leading cause of infant mortality in the United States and are present in approximately 15% of neonates admitted to neonatal or pediatric intensive care units (NICUs/PICUs) [17]. Despite advances in the genetic field, a proportion of children with suspected genetic disorders do not receive a definitive diagnosis [18]. NGS has significantly improved clinical management by promoting more accurate therapeutic decisions and contributing to favorable psychological outcomes for families [19,20,21,22]. However, standard NGS requires up to 6–8 weeks to yield results, increasing the risk of mortality and poor clinical outcomes; a rapid diagnosis is therefore essential to guide timely interventions that can reduce morbidity and mortality [17]. In this context, a rapid genomic technology can deliver faster diagnostic results, enabling precision treatments, avoiding unnecessary interventions, and reducing overall healthcare costs. Prioritizing rNGS is crucial for time-sensitive conditions, especially in neonatal and pediatric intensive care settings. Rapid (r) and ultra-rapid (ur) whole-genome or exome sequencing (WGS/WES), defined by turnaround times of 6–15 days (r) and 1–3 days (ur), enable fast sequencing of a patient’s genome [23]. Recent technological advances allow whole-genome sequencing and analysis in less than 48 h, speeding up clinical decision-making in critically ill newborns [24]. These rapid diagnoses can influence critical healthcare decisions, including possible changes in therapy or surgical interventions. However, the diagnostic yield of NGS varies with disease type; for instance, metabolic and neurologic conditions show higher diagnostic rates than cardiac disorders [25,26]. The fastest reported diagnostic workflow yielded a result in just 7 h and 18 min, with genome sequencing completed in 5 h and 2 min [27]. Thus, the utility of rWGS in critically ill pediatric patients and its integration into routine clinical care have been demonstrated in several studies [28,29,30,31,32,33,34,35,36]. Table 1 summarizes the most relevant studies on the use of rNGS in pediatric emergency care units. In particular, Project Baby Bear was the first state-funded program in the United States to implement rWGS as a first-line diagnostic test for critically ill newborns with suspected rare genetic diseases [37]. To quantify the clinical and financial effects of rWGS, the project enrolled 178 newborns who were admitted to intensive care hospitals and were covered by California’s Medicaid program (MediCal). The results showed a 43% diagnostic yield and changes in clinical management in 31% of diagnosed cases, resulting in an estimated $2.5 million in healthcare cost savings. Another project, named the Newborn Sequencing In Genomic medicine and public HealTh (NSIGHT) investigates the clinical, ethical, legal, and social implications of rapid genomic sequencing compared to standard genetic testing in newborns [38]. The NSIGHT1 sub-study (NCT02225522), which included *n* = 65 infants under 4 months of age from NICUs and PICUs, showed that rWGS increased diagnostic rates and significantly reduced time to diagnosis; indeed, within 28 days, rWGS demonstrated a diagnostic sensitivity of 31%, compared to 3% for standard testing. The NSIGHT2 trial (NCT03211039) further assessed the clinical utility of rapid and ultra-rapid sequencing in *n* = 1248 ICU infants lacking etiologic diagnoses [39]. This was the first randomized trial to systematically evaluate both rWGS and urWGS in this population. Among the participants, 37% were enrolled within 96 h of ICU admission, and 11% received urWGS. The remaining infants were randomized to rWGS or rWES. UrWGS provided the highest diagnostic yield (46%) and the fastest median turnaround time (4.6 days), while rWGS showed superior analytical performance compared to rWES. The findings support the use of urWGS as an effective first-line diagnostic tool in critically ill neonates [16]. Additional studies report comparable outcomes using rNGS in infants with suspected monogenic disorders. One study reported a 51% diagnostic yield with a median time to referral of 3.3 days [40]; another reported a 39% yield, with most diagnoses influencing clinical management [41]; a third study found a 50% yield, with 33% of patients receiving definitive molecular diagnoses that directly affected care [42]. Clinician survey data support the perceived clinical utility of rWES, rWGS, and urWGS, regardless of result positivity (77%).

In conclusion, integrating rNGS technologies into the diagnostic process of pediatric and neonatal illnesses provides a number of advantages, such as:

- Faster diagnosis: rNGS enables timely identification of genetic causes, essential for life-saving interventions [29,43,44].
- Personalized treatment: rNGS facilitates individualized therapy by avoiding ineffective interventions [45].
- Higher diagnostic precision with trio analysis: inclusion of parental DNA increases the ability to detect de novo mutations, distinguish pathogenic from benign variants, and clarify inheritance patterns—essential for genetic counseling [46].
- Genetic counseling support: rNGS in NICUs and PICUs often includes genetic counseling for families, thus helping families understand the genetic basis of disease and the potential implications for future pregnancies [44,47].
- Research benefits: rNGS provides valuable genomic data for rare disease studies, potentially leading to the identification of new molecular targets and to the development of novel therapies [48].
- Cost-efficiency: while initial costs are high, the use of rNGS as a second-tier diagnostic step following standard tests can reduce overall expenses, including shorter ICU stays [44,49,50].

However, several issues limit the wider adoption of rNGS, despite its clinical benefits. One major challenge is the inter-laboratory variability in turnaround times (TATs), which can delay diagnosis and treatment, particularly in urgent contexts such as oncology or rare diseases. This aspect arises from differences in sequencing platforms, automation levels, sample processing workflows, and bioinformatic pipelines, with TATs ranging from a few days to several weeks [48,51]; the lack of standardized performance benchmarks further compromises results reproducibility and comparability across institutions [52]. While centralized testing and external quality assessment (EQA) programs may improve consistency, they introduce concerns around scalability and equitable access, especially in under-resourced settings [44]. Another key limitation is the shortage of specialized, multidisciplinary personnel capable of managing the technical, computational, and clinical demands of rNGS. Indeed, an effective implementation requires collaboration among highly specialized figures like molecular biologists, bioinformaticians, clinical geneticists, and genetic counselors and expertise that is not uniformly available across healthcare systems. Furthermore, the rapid evolution of NGS technologies necessitates continuous training, but structured educational and accreditation pathways remain limited, leading to variability in service quality and data interpretation capacity [53]. Finally, variant interpretation remains a major bottleneck; while the ability to detect genomic variants has greatly improved, assigning clinical meaning remains complex, especially for variants of uncertain significance (VUS). Current interpretation often relies on databases such as ClinVar and gnomAD, which are incomplete and underrepresent diverse populations [54]. The identification of incidental findings and the implications for patient care require careful management. A study in Italy involving *n* = 4067 newborns found that 13% had actionable genetic conditions identified through whole-exome sequencing, emphasizing the need for robust genetic counseling and informed consent processes [55].

The dynamic evolution of variant classification introduces additional ethical and operational challenges, especially given the lack of standardized policies for communicating revised results [56]. Collectively, these barriers, together with the high costs, highlight the need for standardized, scalable frameworks for TATs management, workforce development, and data interpretation to ensure the effective and equitable integration of rNGS into clinical practice. Moreover, ethical and legal issues regarding informed consent, data confidentiality and potential genetic discrimination must also be addressed. In this context, a key concern is genomic privacy, as sequencing data generated in early life is not only deeply personal but also potentially sensitive over a lifetime. The long-term storage and potential sharing of such data introduce risks of data breaches, re-identification, and misuse by third parties. Robust data governance policies and strict access controls are essential to protect the privacy and rights of the individual throughout the lifespan. Another complex area involves consent procedures for newborns, who cannot provide informed consent themselves. In most cases, parents or legal guardians act as decision-makers, but this raises questions about how much risk and future uncertainty they can ethically consent to on behalf of the child, especially when dealing with findings of uncertain significance or predictive results for adult-onset conditions. Models of tiered or dynamic consent [57], as well as frameworks that prioritize the child’s future autonomy, are increasingly being recommended to address these challenges. Finally, the use of advanced genomic diagnostics highlights pressing issues of equitable access. These technologies are often limited to well-resourced hospitals and research centers, creating disparities in access based on geography, socioeconomic status, or healthcare system infrastructure.

## 3. Laboratory POCT: An Important Opportunity for Pediatric Patients

POCT refers to diagnostic tests performed near the patient, aimed at delivering rapid results to support timely clinical decision-making [58]. The National Academy of Clinical Biochemistry (NACB) has established guidelines concerning their use [59] and is endorsed by the World Health Organization (WHO). According to standard definitions, POCTs should comply with the ASSURED criteria: Affordable, Sensitive, Specific, User-friendly, Rapid and robust, Equipment-free, and Delivered to end-users [58]. In accordance with these criteria, POCTs offer faster diagnoses, better outcomes, shorter hospital stays, and reduced blood sample volumes, particularly in critical care settings such as PICUs and NICUs. Their primary goal is to reduce TAT, length of hospital stays (LOS), and healthcare costs while maintaining diagnostic accuracy; they often provide results within minutes or hours, enabling immediate treatment decisions. Furthermore, POCTs reduce reliance on centralized laboratories and trained personnel, making them particularly useful in resource-limited settings [58]. Their integration supports rapid diagnostic decisions across a spectrum of diseases, especially where rapid intervention is essential. With the shift toward more patient-centered care, POCT has evolved into multiplex POCT (mPOCT), which enables the simultaneous detection of multiple analytes from a single sample. These tests combine high sensitivity and specificity using advanced detection technologies such as fluorescent or electrochemical sensors (e.g., microfluidics, lateral flow assays, PCR-based systems) [60]. The principal innovation of mPOCT lies in its high-throughput capability, facilitating rapid and comprehensive diagnostic insights while maintaining ease of use, even by non-laboratory personnel. Also, mPOCT devices span various clinical domains such as infectious diseases, oncology, autoimmune disorders, and metabolic conditions requiring smaller sample volumes. This improves child comfort and compliance, allowing timely and cost-effective clinical management. In the pediatric field, respiratory pathogen panels simultaneously detect viruses such as influenza, respiratory syncytial virus (RSV), and rhinovirus from nasopharyngeal swabs, aiding in the management of respiratory infections [61,62]. Gastrointestinal panels identify bacterial, viral, and parasitic agents from stool samples, supporting rapid diagnosis in acute pediatric gastroenteritis [63]; also, sepsis panels allow an early detection of multiple bacterial and fungal pathogens in blood samples, essential for timely intervention in pediatric sepsis [64,65]. Recent studies highlight promising applications of POCT in neonatal care, particularly in the early detection and management of sepsis. In 2024 Goyal et al. conducted a prospective observational study in a level-III NICU involving *n* = 82 neonates, demonstrating that point-of-care testing of CRP, IL-6, and procalcitonin showed good diagnostic accuracy compared to standard laboratory methods [66]. Similarly, recently Wang et al. evaluated the combined use of monocyte distribution width and procalcitonin, reporting improved sensitivity and specificity for the diagnosis and prognosis of neonatal sepsis [67]. Also, Siraj Nabi et al. compared serum procalcitonin with blood culture results in *n* = 61 neonates, finding high sensitivity (92%) and specificity (83%) for early sepsis detection [68]. Additionally, mPOCTs are available for sexually transmitted infections [69], allergy screening via specific IgE levels [70], and detection of meningitis and encephalitis pathogens from cerebrospinal fluid [71]. Table 2 summarizes several studies on the use and advantages of POCT in the pediatric population.

In addition, another study showed that point-of-care blood culture volume verification, combined with serial clinical observation, significantly reduced unnecessary antibiotic exposure in newborns ≥ 35 weeks of gestation during the first days of life [72].

However, POCTs and mPOCTs display limitations despite their benefits. Compared to traditional laboratory techniques, analytical sensitivity and specificity may be reduced, increasing the risk of false positives or negatives. Furthermore, the reference intervals for POCTs in children are not well established and children’s physiological changes, especially in neonates, together with the dynamic fluctuation of analyte levels during growth, exacerbate the issue. For analytes like C-Reactive Protein (CRP), hemoglobin, and bilirubin, concordance with central laboratory methods is high [73,74,75], whereas discrepancies are more common with electrolytes [73,76,77] and glucose in neonates. A key challenge is the lack of standardization across devices and testing protocols, which can lead to inconsistent and sometimes inaccurate measurements. Neonatal physiology, including small blood volumes and unique biochemical ranges, requires highly sensitive and precise assays, but many POCT devices are calibrated primarily for adult populations, compromising reliability in neonates. Moreover, the absence of universally accepted quality control guidelines and reference standards for neonatal electrolyte and glucose testing exacerbates these issues, leading to potential risks in clinical management [78]. Thus, combining POCTs with analytical laboratory tests can improve diagnostic accuracy and clinical outcomes. Furthermore, other issues arise from the limited diagnostic scope; in fact, such devices often target a narrow range of biomarkers, excluding other equally important biomarkers. Another major challenge is the control of pre-analytical and analytical variables. Considerable variability exists among device manufacturers in terms of analytical methods, acceptable sample types (i.e., whole blood vs. plasma), and calibration standards, which further complicates result comparability. Such inter-device variability can significantly affect clinical interpretation, particularly when patients are transferred between units utilizing different POCT platforms. Other aspects to consider include the storage conditions of the device such as temperature and humidity levels, which can affect reagent stability and the accuracy of results, especially in pediatric settings [3]. Also, pediatric samples may be inadequate or contaminated, compromising the reliability of the test, especially when the sample volume is limited; furthermore, the variability due to differences between clinical settings and operator skills remains crucial. Finally, the lack of standardization can compromise the reliability of results in non-laboratory settings, making training of dedicated personnel essential to minimize errors. In this context, routine audits are essential to monitor compliance, reduce risks, and ensure quality. Addressing these gaps through the harmonization of testing standards, device validation tailored to neonatal populations, and implementation of quality assurance programs is essential to enhance the accuracy and reliability of POCT in neonatal care. Although POCTs can reduce healthcare costs by streamlining diagnostics and minimizing unnecessary testing, their initial implementation costs may limit accessibility in resource-constrained settings. Additionally, regulatory heterogeneity at both national and international levels can affect the standardization, approval, and clinical reliability of POCTs. In conclusion, while the integration of POCTs into clinical practice, particularly in emergency and intensive care settings, offers considerable advantages over centralized testing, their inherent limitations underscore the need for a hybrid diagnostic approach that combines POCTs with conventional laboratory testing to ensure accuracy, reliability, and optimal patient outcomes.

## 4. Artificial Intelligence for Diagnosis of Children’s Diseases

**Figure 1:**
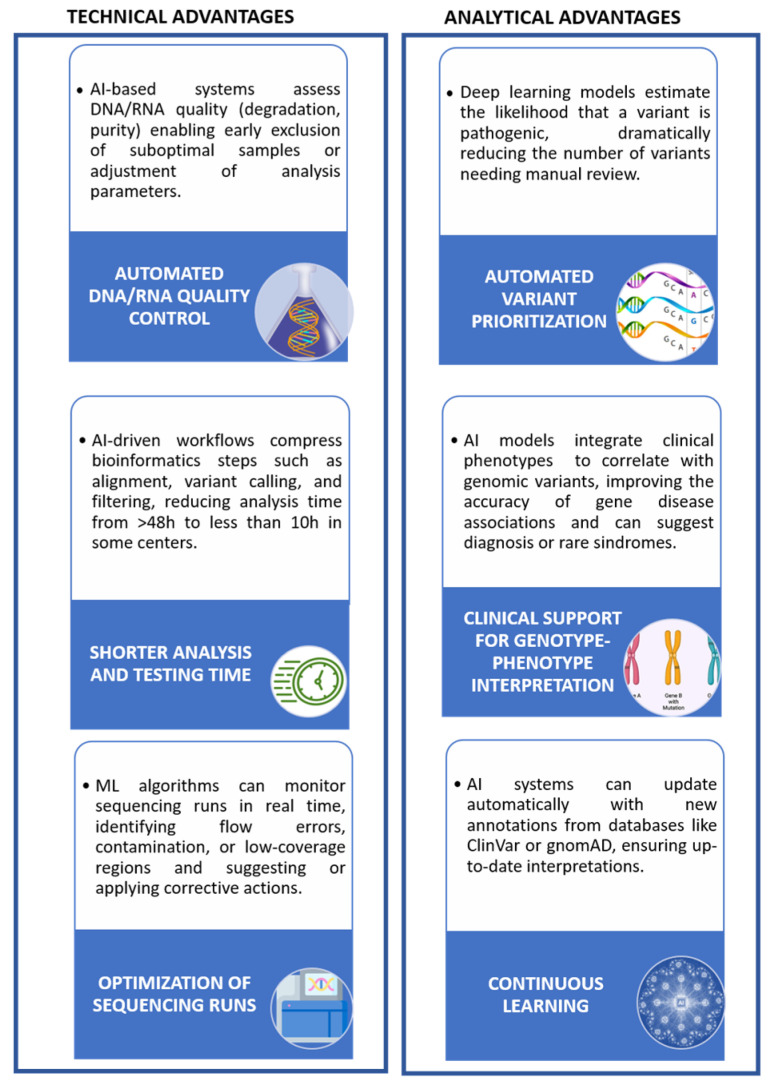
AI in the optimization of rNGS workflow in NICU and PICU settings.

**Figure 2:**
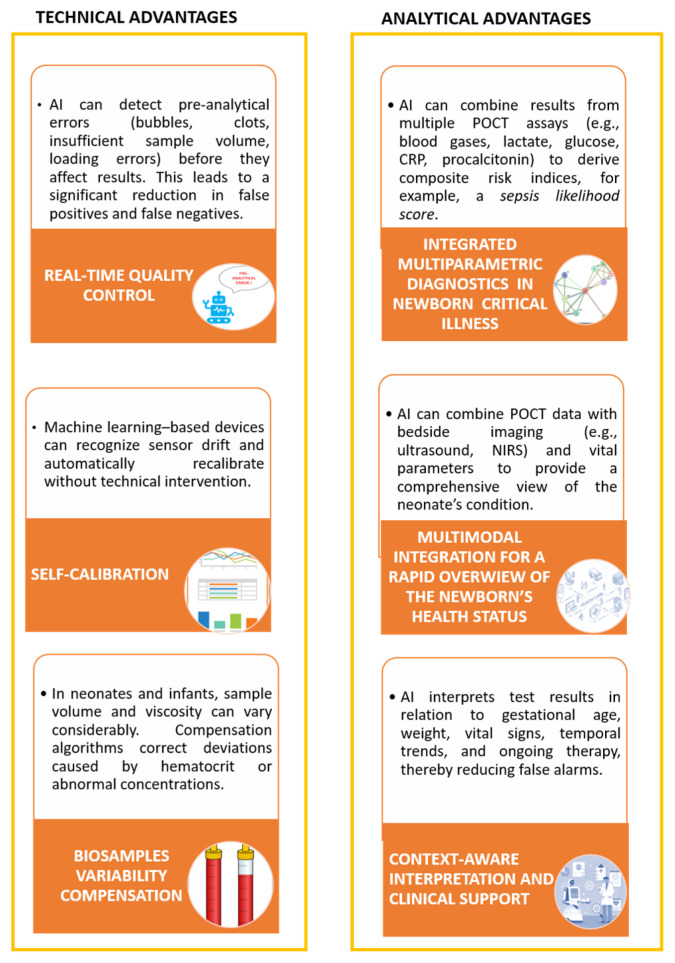
AI in the optimization of laboratory POCT workflow in NICU and PICU settings.

Advances in AI are bringing about significant changes in the field of medical diagnostics, which is currently undergoing a major revolution; AI is introducing innovative tools and solutions that have the potential to improve the accuracy, speed, and effectiveness of diagnostic processes. To this aim, AI algorithms effectively exploit meaningful associations within medical datasets to diagnose, treat, and predict outcomes in different clinical scenarios [79,80]. The workflow automation associated with the management of pediatric biospecimens not only speeds up laboratory operations but also reduces the occurrence of human error, thereby increasing the reliability of results. In this context, AI-based diagnostic algorithms have the potential to transform the way healthcare professionals interpret and use huge amounts of medical data. Medical algorithms can quickly and accurately analyze complex clinical information, facilitating early recognition of disorders, facilitating more accurate diagnoses, and personalizing treatment plans [81]. This increased sensitivity leads to earlier diagnoses, ensuring timely management and better patient outcomes. Table 3 illustrates some of the ways in which AI can improve the diagnosis of pediatric diseases. Furthermore, Figure 1 and Figure 2 provide an overview of the anticipated impact of AI on laboratory improvement and the implementation of rapid NGS in clinical practice.

AI algorithms could play a fundamental role in the elaboration and interpretation of rNGS and POCTs results [90]. First of all, standard diagnostic pipelines are complex and time-consuming, with turnaround times ranging from days to weeks. Especially in rNGS, AI can assist in the swift interpretation of genetic data obtained through sequencing, potentially overcoming the necessity of extensive genetics and bioinformatics expertise required by standard diagnostic pipelines for the diagnosis of genetic diseases, although to date many AI programs have been developed for use by bio-informaticists and geneticists, rather than the clinicians overseeing patient care [91]. By analyzing medical patient records, AI automatically extracts his/her phenotypic information that, in combination with a patient’s genetic data, may reduce the amount of time between DNA sequencing and diagnosis [92,93]. By generating a prioritized list of genetic diseases potentially associated with a patient’s clinical presentation, AI can rapidly provide critical clinical insights. When combined with the speed of rNGS, this approach holds substantial clinical value in neonatal and pediatric intensive care units (NICUs and PICUs), in addition to promoting broader use of exome and genome sequencing. Building on these principles, emerging platforms that integrate CRISPR/RPA-based amplification, deep learning algorithms, and smartphone-assisted analysis have achieved highly sensitive, multiplexed molecular testing in home settings, reaching attomolar detection limits and complete diagnostic concordance across multiple viral targets [94]. When AI is applied to rNGS, the integrated approach could enormously enhance the diagnostic power and the clinical value of the obtained results. In fact, a faster data analysis in succession of a faster laboratory medicine test is the best goal when an urgent medical decision must be taken. In this context, a recent study explored the ability of an open-source AI tool designed for use by non-expert users to analyze data from rNGS on infant admitted to the intensive-care units for suspected Mendelian disorders. Results clearly show how the specific AI tool required less than 1h for the fully automated analysis, from data upload to generation of the final differential diagnosis, helping clinicians to quickly identify a manageable list of potential diagnoses among genetic diseases [15]. AI has innovative and useful capability in several pre- and post-analytical variables, automating clinical laboratories, enhancing quality control, improving testing efficiency, and playing a generally critical role in streamlining laboratory workflows [95] by continuously monitoring POCT device performance, ensuring that tests remain reliable, and that maintenance is performed promptly. This proactive approach to quality control reduces the risks associated with test errors. Such AI features could power POCT devices and the entire pipeline from sample collection to results [96]. Furthermore, AI facilitates the interpretation of data generated by mPOCTs by identifying correlations and anomalies that may be overlooked by human analysis leading to a more accurate and timely diagnosis. In the case of visual investigations, such as immunoassays or microscopy, AI-based image detection technologies can automate the analysis, improving the sensitivity and reducing human bias. Recent progress in AI-driven microfluidics is revolutionizing point-of-care diagnostics and decentralized molecular testing by combining automation, miniaturization, and intelligent data processing. The integration of AI with microfluidic systems, which are transforming the landscape of POCT, allows the creation of fully automated “sample-in, answer-out” diagnostic workflows for rapid diagnosis by enhancing chip design and streamlining sample handling, by improving signal interpretation and enabling real-time feedback control [97]. Other AI-powered systems integrate machine vision with multicolor lateral flow assays, providing quantitative and accessible results for pathogens such as Escherichia coli and SARS-CoV-2 directly through mobile interfaces [98]. In parallel, encoded microsphere biosensors decoded through deep-learning algorithms enable multiplex biochemical analysis of proteins, bacteria, viruses, and antibiotics with exceptional precision and portability [99]. Further innovations include AI-enhanced Lab-on-a-Disc devices for rapid and multiplex identification of biological thiols, capable of distinguishing structurally similar compounds with 100% accuracy from minimal blood samples within minutes [100]. Deep-learning-assisted microfluidic immunoassays have also been developed for simultaneous detection of respiratory viruses and antibiotics, combining microsphere-based encoding–decoding strategies with smartphone imaging for rapid quantification [101].

Collectively, these advances demonstrate how the convergence of AI, microfluidics, and mobile technologies is transforming modern diagnostics—offering fast, accurate, and affordable testing that supports personalized medicine, continuous epidemic surveillance, and enhanced global public health resilience.

Another advantage of AI is its ability to provide predictive analyses by integrating data with a patient’s medical history and identifying patients at risk, thereby optimizing treatment strategies. This aspect also connects to tailored medicine, where mPOCT data can be combined with genetic, environmental, and lifestyle factors to develop highly personalized treatment plans, which are particularly valuable in pediatric care. Furthermore, AI can improve efficiency by automating tasks, thus freeing up time for healthcare professionals to focus on patient care. Despite their potential, the integration of AI in pediatrics presents several challenges. One of the most pressing needs in the implementation of AI in pediatric settings is the establishment of robust clinical validation frameworks. While many AI models demonstrate high performance in retrospective datasets, prospective validation, particularly in multicenter pediatric populations, is often lacking. Pediatric patients differ significantly from adults in physiology, disease progression, and treatment response, making age-specific validation essential. Furthermore, for pediatric applications validation studies must also account for age stratification (e.g., neonates vs. adolescents), differences in clinical presentation, and developmental variability. For the safe inclusion of pediatric data in AI and machine learning, it is crucial to protect children’s health data and address the fundamental parameters of age, consent, communication, and equity. Robust data encryption and compliance with privacy regulations are necessary. Furthermore, AI algorithms present several biases that could make healthcare outcomes not equitable, especially in the pediatric population, and must undergo rigorous clinical validation to demonstrate their safety, efficacy, and reliability in the context of age-specific populations. In this regard, a well-documented issue is the underrepresentation of pediatric and neonatal data in many clinical datasets, especially from diverse ethnic, socioeconomic, and geographic populations. This leads to algorithmic bias, where model predictions may be less accurate for subgroups not adequately represented in the training data [102]. At the same time, the underrepresentation of children, especially from diverse backgrounds, in training datasets can introduce bias and compromise the fairness and accuracy of AI-supported decisions. Finally, regulatory frameworks must ensure the safety, transparency, and adaptability of these tools, with specific requirements for pediatric use and mechanisms for ongoing performance monitoring.

It is crucial to address ethical, privacy, and regulatory considerations to ensure AI in line with advocacy and safeguarding children [103]. The regulatory landscape for AI in healthcare is still developing, with specific challenges for pediatric applications. Agencies like the FDA and EMA have begun addressing AI-specific issues through frameworks such as the FDA’s Action Plan for AI/ML-based Software as a Medical Device, which promotes a lifecycle approach to regulation. For pediatric AI tools, it is essential to ensure evidence of safety and efficacy in children not extrapolated from adult data, along with mechanisms for model adaptability, revalidation, and transparency in design and use. Regulatory oversight must also balance innovation with patient safety, particularly in critical settings like NICUs, where AI may assist in detecting medical conditions. Overall, the integration of AI into pediatrics, especially in the domains of rNGS and POCTs, holds the potential to revolutionize pediatric healthcare by enabling faster and more accurate diagnoses, personalized treatment approaches, and improved overall care for children [104]. However, current evidence remains insufficient to demonstrate that AI has substantially improved health outcomes in pediatric critical care. To accurately assess AI’s clinical impact, additional prospective and experimental studies are required, employing validated outcome measures, standardized performance metrics, and well-defined implementation frameworks [105]. Most AI models developed for NICU and PICU remain at the testing or prototyping stage and are characterized by a high risk of bias. Bridging the gap between model design and clinical implementation is essential to ensure the development of safe, reliable, and clinically meaningful AI tools. The establishment of specific guidelines and methodological approaches may further support the integration of AI into clinical practice, ultimately improving patient outcomes [106].

Furthermore, ethical, juridical and technical aspects need to be more defined for the introduction of AI into the clinical practice of pediatrics.

## 5. Challenges, Strategies, and Stakeholder Roles in the Implementation of rNGS, POCT, and AI in NICU and PICU

**Figure 3:**
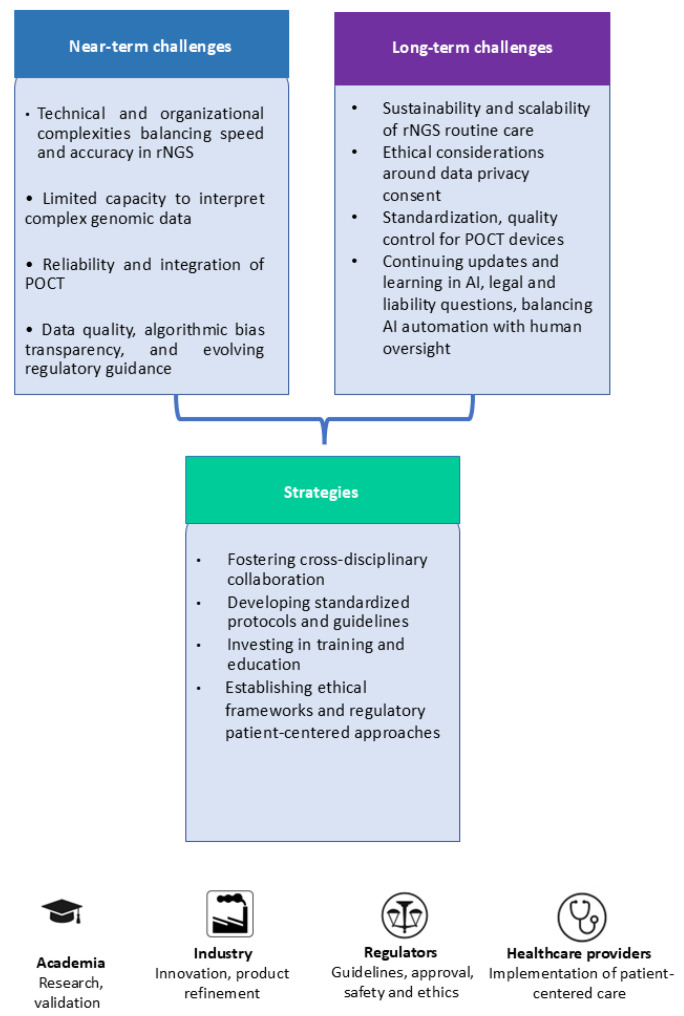
Schematic overview summarizing near-term and long-term limitations, operational frameworks, and principal stakeholders involved in the integration of rNGS, POCT, and AI technologies within pediatric and neonatal diagnostics.

The use of rNGS, POCT and AI in pediatric care, particularly neonatal care, presents both near-term and long-term challenges. Near-term challenges include technical and organizational complexities such as balancing speed and accuracy in rNGS for time-sensitive neonatal care, limited clinical capacity to interpret complex genomic data, ongoing concerns about the reliability and integration of POCT in routine workflows, and persistent issues in AI related to data quality, algorithmic bias, limited transparency, and evolving regulatory guidance. Long-term challenges involve the sustainability and scalability of rNGS in routine care, ethical considerations around data privacy and consent, standardization and quality control for POCT devices, ongoing training requirements, the need for continual updates and learning in AI systems, legal and liability questions, and balancing AI automation with human oversight. To overcome these challenges, potential strategies include fostering cross-disciplinary collaboration, developing standardized protocols and guidelines, investing in comprehensive training and education, establishing clear ethical frameworks and regulatory pathways tailored to pediatrics, and enhancing healthcare infrastructure for seamless integration of these technologies. Different stakeholders play critical roles in addressing these issues: academia leads in research, validation, and education; industry drives innovation, product refinement, and regulatory collaboration; regulators provide guidelines, approvals, and oversight on safety and ethics; and healthcare providers implement these technologies within clinical practice while advocating for patient-centered, equitable care. Together, these efforts aim to optimize the benefits of rNGS, POCT, and AI in pediatric and neonatal care, ensuring safe, effective, and ethical application. Figure 3 summarizes challenges, strategies, and stakeholder roles.

## 6. Discussion

The combination of cutting-edge technological tools, such as rapid NGS, with AI-driven POCT devices and diagnostic software represents a major advancement in modern laboratory medicine. Indeed, in ICU settings, the integration of POCT with AI-driven deep learning models has markedly improved sepsis prediction, enabling earlier administration of antibiotics and reducing mortality by up to 18% [107]. AI-powered POCT systems have also enhanced emergency triage by merging rapid antigen test results with real-time patient vital signs, achieving an AUC-ROC of 0.89 for high-risk patient stratification and decreasing time-to-isolation by 40%, ultimately improving clinical outcomes [108]. These examples highlight the ability of AI to integrate heterogeneous data, from molecular biomarkers to clinical records, and transform them into actionable insights that support diagnostic accuracy and clinical decision-making, as recently emphasized by T.S. Pillay et al. [109]. Specifically, the implementation of POCT in emergency units such as NICUs and PICUs holds significant promise for improving the quality of care and patient survival by streamlining diagnostic workflows, shortening time to intervention, reducing hospital stays, and lowering healthcare costs [110]. Furthermore, when supported by appropriate staff training and education, POCT adoption has been linked to improved workflow efficiency, greater staff satisfaction, and further administrative and economic benefits. However, the majority of available evidence on POCT performance derives from adult populations, and its clinical utility may differ substantially in children. For instance, pediatric hyperglycemia associated with metabolic acidosis is more frequently a manifestation of systemic inflammation rather than diabetic complications, which are more common in adults. These differences underscore the need to tailor diagnostic algorithms and clinical decision-making frameworks specifically to the pediatric patients [111]. In recent years, advances in lab-on-a-chip technologies and AI-driven analytical systems have shown a great potential to enhance the precision, reliability, and operational efficiency of POCT in critical care settings. Nonetheless, current POCT devices still face important limitations. Some assays exhibit high specificity but limited sensitivity, which can result in false-positive findings, as occasionally seen with nasal swab tests for respiratory pathogens such as influenza [112]. To address these shortcomings, the integration of POCT with rNGS has emerged as an effective complementary strategy.

The combined use of POCT and rNGS can substantially improve diagnostic accuracy, thereby enabling more informed clinical decision-making and optimized patient management. Notably, rapid NGS has now reached a level of technological maturity that supports routine clinical deployment within critical care services in several countries, including the United Kingdom, Australia, and the United States. In England, for instance, the National Health Service (NHS) provides government-funded genomic testing programs that incorporate rapid NGS into clinical workflows. Since October 2019, the NHS has specifically adopted rWES for critically ill pediatric patients. This strategy has significantly shortened diagnostic turnaround times, enabling the identification of genetic disorders within hours to a few days instead of several weeks, with reported diagnostic yields of approximately 36% in critically ill neonates [27,40,48]. However, given the high level of specialization required, it is essential that healthcare professionals possess a comprehensive understanding of the testing strategies employed, the range of detectable variants, and the associated ethical implications. Knowledge gaps among non-genetics healthcare providers remain evident [113]; therefore, structured training programs are critical to support appropriate test selection and effective clinical management from sample acquisition to result interpretation. In the United Kingdom, genomic education is now incorporated into medical school curricula and coordinated by the Genomic Education Programme of Health Education England [114]. Furthermore, the clinical implementation of rNGS still requires streamlined logistics, standardized bioinformatics pipelines, and rapid variant interpretation frameworks, which can be strengthened using AI-assisted annotation tools that prioritize pathogenic variants while maintaining transparency and reliability [15,80]. Nevertheless, some NGS providers like Illumina (San Diego, CA, USA) endeavored to mitigate this limitation through the development of DRAGEN, AI-enabled variant-calling and interpretation platform widely used for clinical prioritization. Its computational approach integrates called variants, pathogenicity predictions, and Human Phenotype Ontology (HPO)-based annotations to generate a curated list of candidate variants most likely to explain the observed phenotype [115]. Furthermore, when variants are novel or not represented in established databases, AI-based predictive modeling can assist in assessing their potential effects on protein structure, function, or RNA splicing, thereby improving diagnostic accuracy in complex or previously uncharacterized cases [116,117].

**Figure 4:**
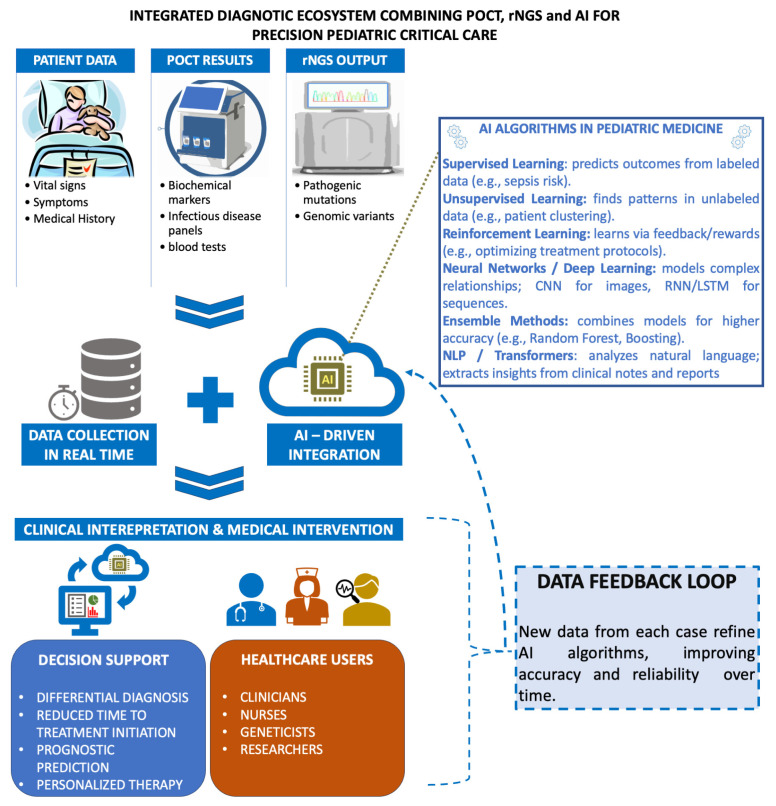
AI-driven diagnostic ecosystem for the real-time integration of patient data as well as POCT and rNGS results for pediatric critical care. Over time AI algorithms will improve from each case, and new data will refine accuracy and reliability.

Modern healthcare technologies achieve their greatest clinical impact when used in combination, generating synergistic improvements in patient management—particularly in neonatal and pediatric critical care. When rapid genetic testing is combined with POCT platforms, clinicians can perform genetic analyses directly at the patient’s side, drastically shortening turnaround times and bringing a critical advantage in neonatal and pediatric intensive care. This combined approach facilitates accelerated differential diagnosis by merging laboratory and clinical data through AI-based analytical systems. As illustrated in Figure 4, such integration establishes a comprehensive diagnostic ecosystem in which patient information, POCT findings, and rNGS results are continuously aggregated and processed using AI infrastructure. This supports multiple healthcare stakeholders, including clinicians, nurses, geneticists, and researchers, and enhances coordinated clinical decision-making. A major paradigm shift is driven by this convergence of data streams: rapid NGS contributes genomic profiling, POCT provides real-time biochemical and physiological data, and AI performs multidimensional interpretation, ultimately transforming heterogeneous inputs into actionable diagnostic insights [90,108].

Within this integrated framework, AI not only accelerates variant prioritization but also contextualizes genomic discoveries alongside phenotypic and clinical information, thereby generating more actionable insights; this supports hybrid diagnostic workflows that enable earlier disease recognition, optimize therapeutic timing, and reduce unnecessary interventions in NICU and PICU environments [118]. Additionally, AI-driven analytics can process POCT results in real time to improve diagnostic accuracy, minimize human error, and deliver immediate decision support at the bedside without dependence on centralized laboratory infrastructure. At the same time, AI facilitates rapid interpretation of large-scale genetic data, advancing precision therapy for critically ill pediatric patients requiring urgent intervention.

Despite these technological advances, significant ethical, regulatory, and infrastructural challenges remain. Pediatric data management requires age-appropriate consent processes, rigorous anonymization strategies, and safeguards to preserve the child’s future autonomy [119]; additionally, many AI systems are trained predominantly on adult datasets, raising concerns of algorithmic bias and variable performance across pediatric subgroups [102]. Regulatory agencies such as the EMA and FDA have begun establishing pediatric-specific validation frameworks to ensure model safety, fairness, and explainability [120,121].

Economic considerations are also critical; although rapid genetic testing and AI-based POCT entail considerable upfront investment, these costs may be counterbalanced by reductions in ICU stays, avoidance of unnecessary procedures, and more precise, timely treatments yielding measurable benefits for families and healthcare systems [122]. Broader adoption is further hindered by limited funding, infrastructural disparities, and the shortage of interdisciplinary professionals capable of integrating genomic, laboratory, and computational data. Looking ahead, to fully realize the potential of rNGS, POCT, and AI in pediatric critical care, will require coordinated action at institutional, national, and international levels. Key priorities include sustained investments in healthcare infrastructure to support acquisition, maintenance, and seamless integration of advanced diagnostic technologies within clinical workflows. Such investments must prioritize equitable access, ensuring that resource-limited settings are not left behind in the genomic and digital medicine revolution. Equally critical is the development of interdisciplinary training programs that bridge clinical, laboratory, and computational expertise. Empowering healthcare professionals, including neonatologists, nurses, geneticists, bioinformaticians, and data scientists, with the knowledge and skills needed to interpret and act on complex diagnostic data will enhance the accuracy, efficiency, and clinical utility of these technologies. Furthermore, scalable implementation strategies should emphasize harmonization of standards and protocols, robust clinical validation, and regulatory frameworks tailored to pediatric populations. Leveraging telemedicine and cloud-based platforms can facilitate remote support and data sharing, fostering collaboration across institutions and geographic regions. By adopting these comprehensive approaches, healthcare systems worldwide can accelerate the safe and effective integration of POCT, rNGS, and AI, ultimately improving diagnostic precision, patient outcomes, and personalized care in NICU and PICU settings.

## 7. Conclusions and Future Outlook

Moving forward, we believe that for achieving a full integration of rNGS, POCT, and AI in pediatric critical care will depend on several key elements:

▪ Cross-disciplinary education, fostering novel professional roles such as hybrid clinician–data scientists who can bridge medical expertise with computational and analytical proficiency.
▪ Explainable AI tools designed for non-specialists, enabling clinicians without advanced data science training to interpret AI-generated insights, critically evaluate algorithmic outputs, and incorporate them into real-time clinical decision-making.
▪ Dynamic ethical governance that evolves in parallel with technological progress, ensuring that frameworks for consent, data sharing, algorithmic accountability, and patient privacy remain adaptive, transparent, and responsive to rapid developments in genomic and AI-driven diagnostics.
▪ Global harmonization of diagnostic standards and neonatal/pediatric data frameworks, promoting interoperability across institutions and countries, improving data quality and reproducibility, and ensuring equitable access to high-quality genomic and clinical resources for all pediatric populations.

When implemented under responsible governance, AI-driven clinical data interpretation has the potential to be a transformative resource for healthcare professionals, accelerating treatment initiation in urgent cases and simultaneously advancing the continuous knowledge of childhood diseases. In this scenario, national healthcare systems needs to make strategic investment in digital and laboratory infrastructures, especially in the case of pediatric populations where reference value are often missing or derived from adult population. Only through such coordinated and ethically guided efforts can the full potential of technological innovation be realized, translating precision diagnostics into meaningful clinical benefit for critically ill children.

## Figures and Tables

**Table 1:** Summary of the major studies assessing the clinical utility of rapid genomic sequencing in critically ill neonates and infants, including study populations, sequencing methods, and main outcomes related to diagnostic yield, turnaround time, clinical impact, and cost-effectiveness.

Study/Project	Population	Intervention/Method	Main Results	References
Baby Bear	*n* = 178 critically ill newborns covered by Medicaid	rWGS as a first-line diagnostic test	43% diagnostic yield, changes in clinical management in 31% of diagnosed cases, $2.5 million healthcare cost savings	[37]
NSIGHT1 (NCT02225522)	*n* = 65 infants under 4 months from NICU/PICU	rWGS vs. standard genetic testing	31% diagnostic yield within 28 days for rWGS vs. 3% for standard testing	[38]
NSIGHT2 (NCT03211039)	*n* = 1248 critically ill ICU infants	Randomized rWGS, urWGS, and rWES	urWGS had the highest diagnostic yield (46%) and the fastest median turnaround time (4.6 days); rWGS outperformed rWES	[39]
Australian Genomics Acute Care program	*n* = 450 infants with suspected monogenic disorders	rWGS or rWES	51% diagnostic yield, median referral time 3.3 days	[40]
NIHMS1870862	*n* = 75 infants with suspected monogenic disorders	rNGS	39% diagnostic yield, most diagnoses influenced clinical management	[41]
Pilot study (Acibadem Mehmet Ali Aydinlar University)	*n* = 10 infants with suspected monogenic disorders	rNGS	50% diagnostic yield, 33% of patients received definitive molecular diagnoses directly impacting care	[42]

**Table 2:** Summary of studies on POCT applications in pediatric and neonatal diagnostics.

Authors	Target	Population	Intervention/Method	Main Results	References
Donà D et al., 2025, Bellini T et al., 2024	Respiratory pathogen panels	Pediatric patients	Multiplex POCT detecting influenza, RSV, rhinovirus	Facilitates management of respiratory infections	[61,62]
Kanwar N et al., 2023	Gastrointestinal panels	Pediatric patients	Multiplex POCT detecting bacterial, viral, parasitic agents	Rapid diagnosis of acute pediatric gastroenteritis	[63]
Tamelytė E et al., 2019, Teggert A et al., 2020, Goyal M et al., 2024, Wang W et al., 2025, Nabi S et al., 2025	Sepsis panels	Pediatric patients	Multiplex POCT detecting bacterial/fungal pathogens in blood	Early detection of sepsis crucial for timely intervention	[64,65,66,67,68]
Adamson PC et al., 2020	sexually transmitted disease panels	Pediatric /adolescent patients	Multiplex POCT	Supports rapid diagnosis of sexual illnesses	[69]
Demuru S et al., 2024	Allergy panels	Pediatric patients	POCT measuring specific IgE levels	Useful for allergy diagnostics	[70]
Tsao YT et al., 2020	Meningitis/encephalitis panels	Pediatric patients	POCT detecting pathogens in cerebrospinal fluid	Rapid diagnosis critical for management	[71]

**Table 3:** How AI can improve diagnosis of pediatric diseases.

Application	Description	Benefits	Pathologies	Doi
Diagnostic Support	AI algorithms analyze medical data to assist in diagnosing conditions in children.	Faster and more accurate diagnoses.	Asthma	[82] 10.21037/atm-20-2501a
Predictive Analytics	Predicts potential health issues based on patient data and trends.	Early intervention and prevention strategies.	Obesity	[83] 10.4103/jfmpc.jfmpc_469_23
Personalized Treatment Plans	Tailors treatment plans based on genetic, environmental, and lifestyle factors.	More effective and individualized care.	Cancer, Chronic Conditions	[84] 10.37349/etat.2023.00127
Telemedicine and Virtual Care	AI enhances remote consultations and monitoring through virtual platforms.	Improved access to care, especially in rural areas.	ADHD	[85] 10.3389/fpsyt.2023.1164433
Radiology and Imaging Analysis	Automates the analysis of pediatric imaging, such as X-rays and MRIs.	Reduced workload for radiologists and faster results.	Tumors	[86] 10.1007/s11604-023-01437-8
Medication Management	AI helps in optimizing medication dosages and schedules for pediatric patients.	Reduces medication errors and enhances safety.	Epilepsy	[87] 10.1001/jamaneurol.2023.1645
Patient Monitoring	Continuous monitoring of vital signs and health data through AI algorithms.	Early detection of potential complications.	Heart Conditions	[88] 10.3390/jcm11237072
Mental Health Support	AI-driven chatbots and applications provide mental health resources for children.	Increases access to mental health support.	Depression, Anxiety Disorders	[89] 10.1186/s13034-023-00586-y

## Data Availability

Data are contained within the article.
